# A Lightweight Remote Sensing Payload for Wildfire Detection and Fire Radiative Power Measurements

**DOI:** 10.3390/s23073514

**Published:** 2023-03-27

**Authors:** Troy D. Thornberry, Ru-Shan Gao, Steven J. Ciciora, Laurel A. Watts, Richard J. McLaughlin, Angelina Leonardi, Karen H. Rosenlof, Brian M. Argrow, Jack S. Elston, Maciej Stachura, Joshua Fromm, W. Alan Brewer, Paul Schroeder, Michael Zucker

**Affiliations:** 1 Chemical Sciences Laboratory, National Oceanic and Atmospheric Administration, Boulder, CO 80305, USA; 2 Cooperative Institute for Research in Environmental Sciences, University of Colorado Boulder, Boulder, CO 80309, USA; 3 Smead Aerospace Engineering Sciences, University of Colorado Boulder, Boulder, CO 80309, USA; 4 Black Swift Technologies, LLC, Boulder, CO 80301, USA; 5 NRC Research Associateship Programs, Washington, DC 20001, USA

**Keywords:** wildfires, fire radiative power, fire temperature, fire mapping

## Abstract

Small uncrewed aerial systems (sUASs) have the potential to serve as ideal platforms for high spatial and temporal resolution wildfire measurements to complement aircraft and satellite observations, but typically have very limited payload capacity. Recognizing the need for improved data from wildfire management and smoke forecasting communities and the potential advantages of sUAS platforms, the Nighttime Fire Observations eXperiment (NightFOX) project was funded by the US National Oceanic and Atmospheric Administration (NOAA) to develop a suite of miniaturized, relatively low-cost scientific instruments for wildfire-related measurements that would satisfy the size, weight and power constraints of a sUAS payload. Here we report on a remote sensing system developed under the NightFOX project that consists of three optical instruments with five individual sensors for wildfire mapping and fire radiative power measurement and a GPS-aided inertial navigation system module for aircraft position and attitude determination. The first instrument consists of two scanning telescopes with infrared (IR) channels using narrow wavelength bands near 1.6 and 4 µm to make fire radiative power measurements with a blackbody equivalent temperature range of 320–1500 °C. The second instrument is a broadband shortwave (0.95–1.7 µm) IR imager for high spatial resolution fire mapping. Both instruments are custom built. The third instrument is a commercial off-the-shelf visible/thermal IR dual camera. The entire system weighs about 1500 g and consumes approximately 15 W of power. The system has been successfully operated for fire observations using a Black Swift Technologies S2 small, fixed-wing UAS for flights over a prescribed grassland burn in Colorado and onboard an NOAA Twin Otter crewed aircraft over several western US wildfires during the 2019 Fire Influence on Regional to Global Environments and Air Quality (FIREX-AQ) field mission.

## 1. Introduction

Wildfires significantly impact air quality on local, regional and even continental scales and may play an interactive role in climate change through emissions and changes in carbon sequestration and storage [1,2]. Since the mid-1980s, the typical annual wildfire area across the western United States has doubled, attributed in part to increased temperatures and decreased moisture due to climate change [3,4]. The climate trend in the western U.S. toward warmer and drier conditions is projected to continue [5]. This trend, combined with the accumulated combustible biomass due to fire-control practices over the past century, suggests that the frequency and scale of wildfires will continue to increase in coming decades, with negative impacts on air quality and health. Similar trends have occurred and are predicted to continue for other fire-prone temperate and boreal regions globally [6,7,8,9].

Satellite measurements of wildfires are used extensively for fire detection and monitoring. Fire radiative power (FRP), a measure of fire intensity, is derived from wavelength bands near 4 µm from MODIS (Moderate Resolution Imaging Spectroradiometer) instruments on the NASA Terra and Aqua satellites and VIIRS (Visible Infrared Imaging Radiometer Suite) instruments on NOAA satellites [10,11]. As an indication of fire intensity, FRP is used as a model input for fire smoke forecasting (e.g., [12]). The spatial resolutions of these satellite instruments are typically several hundred meters. Even though wildfires may burn extensive areas over their lifetimes, active fire areas at any given time are frequently substantially smaller than MODIS (1 km) and VIIRS (375 m) pixel sizes. Therefore, the FRP as seen by individual satellite pixels is typically an average of the active fire and surrounding (much cooler) areas. As a result, FRP measurements from these instruments may not have a direct and monotonic relationship with actual FRP of the flaming regions that are related to emissions and dynamics. FRP measurements at higher spatial resolutions are available from airborne instruments such as the Enhanced MODIS Airborne Simulator (eMAS; ref. [13]) and the MODIS/ASTER Airborne Simulator (MASTER; ref. [14]). Size and weight limit the use of these instruments to larger research aircraft such as King Air B200s and the NASA ER-2 and DC-8.

Uncrewed aerial systems (UASs) have the potential to serve as ideal platforms for wildfire measurements to augment satellite observations with higher resolution data. With no human onboard, they remove the aircrew safety issue associated with operating crewed aircraft in hazardous environments and provide a capability for high resolution measurements in the near-fire environment. There have been a number of demonstrations of small and medium-sized UAS applications for wildfire reconnaissance over the past several years (e.g., [15,16]). These demonstrations have shown that UAS observations can indeed provide useful information for wildfire incident response efforts by accurately detecting fire perimeter and identifying fire hotspots, but there have so far been no attempts to make measurements relevant to studying fire emission behavior or to use the observations as input for fire weather forecast models. A recent review of early forest fire detection approaches, including UAS-based systems, is presented by Barmpoutis et al. [17].

A wildfire’s intensity and emissions depend on a number of factors, including terrain, fuel type, fuel moisture and meteorological conditions. The interaction with local meteorology (winds, temperature and humidity) means that fire emissions typically exhibit a significant diurnal cycle [18]. Fire plumes tend to be more concentrated at night due to reduced mixing and lower boundary layer heights, which degrades air quality leading to higher pollution exposures relative to daytime in areas directly downwind of fires. Differences in winds and surface heating between day and night lead to differences in the rate and direction of fire spread. Manned research aircraft flights are mostly restricted to daytime operations due to potential dangers associated with nighttime operations. This limitation leaves a large data gap in observations of fire perimeter, fire intensity, fire emissions, plume distribution and meteorological data that a capable UAS observation system could fill, allowing for improvements in fire classification [19] and fire weather forecasting as well as providing updated information to local incident management teams (IMT) coordinating fire response efforts.

Recognizing the potential utility of small UASs for wildfire measurements, the National Oceanic and Atmospheric Administration (NOAA) Uncrewed Systems Research Transition Office, formerly known as the NOAA UAS Program Office, funded the Nighttime Fire Observations eXperiment (NightFOX) project to develop and deploy lightweight, low power UAS payload instrument suites for remote sensing of wildfire properties and for in situ smoke plume characterization. Here we present a novel wildfire remote sensing instrument suite developed as part of the NightFOX project. The payload was originally designed for high spatial resolution FRP measurement and fire perimeter mapping (Section 2) while operated onboard a small fixed-wing UAS, but is easily adaptable for measurement from a variety of platforms such as crewed aircraft or rotary-wing UAS. Results from deployments of this sensor suite on a crewed aircraft and a fixed-wing UAS are presented in Section 3.

## 2. Materials and Methods

### 2.1. General System Design Considerations

While the NightFOX remote sensing instrument suite has been successfully adapted for use on manned aircraft, its initial design for use on a small UAS required that the system be compact, lightweight, relatively inexpensive and have a power consumption low enough that a small rechargeable battery pack could be used to power the payload for several hours of continuous operation. The system described here has been designated as the Airborne Wildfire Spectral Mapper (AWSM). The AWSM system sensors were designed to produce continuous spatial coverage for fire mapping and FRP measurement when operated at an altitude of 1000 m above ground at flight speeds less than 20 m s^−1^.

### 2.2. System Configuration

The AWSM system consists of three optical instruments with five individual sensors (two scanning telescopes and three cameras) for fire observations and a GPS-aided inertial navigation system (GPS/INS; VN-200, VectorNav, Dallas, TX, USA) for high frequency aircraft position and attitude determination. The first optical instrument is a cross-track scanner with two narrowband IR telescopes (4 µm and 1.6 µm) for FRP measurements. The second instrument is a broadband shortwave infrared (SWIR) camera (0.95–1.7 µm) for active fire mapping. Its wavelength range encompasses the narrow 1.6 µm band used by the 1.6 µm telescope, and therefore may be used to validate the telescopes’ data mapping algorithm. Both of these instruments are custom built. The third instrument is a commercial off-the-shelf visible/thermal IR dual camera for smoke and flame (visible band) and fire (thermal band) identification and mapping. Various system components were selected based on a range of factors including performance, size, weight, power required, availability and price.

The AWSM system design concept is to use the two scanner channels for calibrated FRP measurements with a 1° field of view (FOV), and the SWIR camera for higher resolution fire extent measurements. The thermal camera, sensitive to temperatures at or above ambient, is suitable for identifying recently burned areas and smoldering combustion. The visible camera records flames, and, during daytime, smoke plumes and the overall environment.

### 2.3. Two-Channel IR Scanner

**Figure 1:**
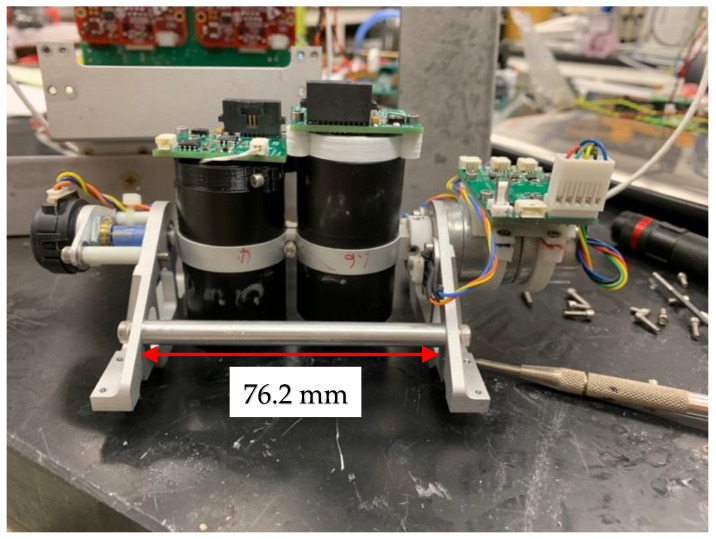
The AWSM two-channel infrared scanner. The sensor telescopes are scanned back and forth across the aircraft flight path, driven by a servo motor (**right** side). An angular encoder (**left** side) measures the telescope pointing angle relative to aircraft nadir.

The two channels in the IR scanner instrument consist of single-element sensors mounted in optical telescopes (Figure 1) and are designed to detect fire and measure FRP. One channel measures FRP in the mid-wavelength infrared (MWIR) near 4 µm (3.925–3.995 µm), which closely matches the fire detection bands (21 and 22, 3.929–3.989 µm) of the MODIS satellite instrument and is similar to the M13 (3.97–4.13 µm) and I4 (3.55–3.93 µm) fire detection bands of VIIRS. The second scanner channel measures FRP in the SWIR near 1.6 µm (1.580–1.640 µm), which matches the VIIRS I3/M10 bands that have been used to detect and characterize oil well flaring. The two telescopes are scanned synchronously across the flight path at approximately 1 Hz (1 scan back and forth per second). Signals from both sensors are digitized at 1° intervals throughout the scan. The scan angle range in the described configuration is approximately ±58° from nadir, however the sensor views are blocked by the UAS nose cone or aircraft skin at large scan angles. As a result, only data with scan angles within ±30° of nadir are used for mapping and FRP determination, while data at large angles are used for monitoring sensor background signal levels. Both scopes have a 1° FOV, which yields a horizontal measurement scale of 0.0175–0.0230 × altitude, resulting in an 18–23 m diameter footprint when viewing from 1000 m above ground level (AGL) and with the 1° sampling interval provides effectively continuous spatial coverage at ground speeds of less than 20 m s^−1^. The scan angle range is set by a 3D-printed cam, which can be replaced to adjust the scan range as desired for other configurations.

A primary reason for using scanning telescopes instead of wide angle, multi-element sensors (cameras) for the FRP measurements is to ensure a consistent wavelength band at all viewing angles. Narrowband optical filters perform as specified by manufacturers only when the incident light is perpendicular to the filter surfaces. It is impossible to use a single conventional filter to achieve uniform wavelength bandpass over the wide FOV of a camera.

**Figure 2:**
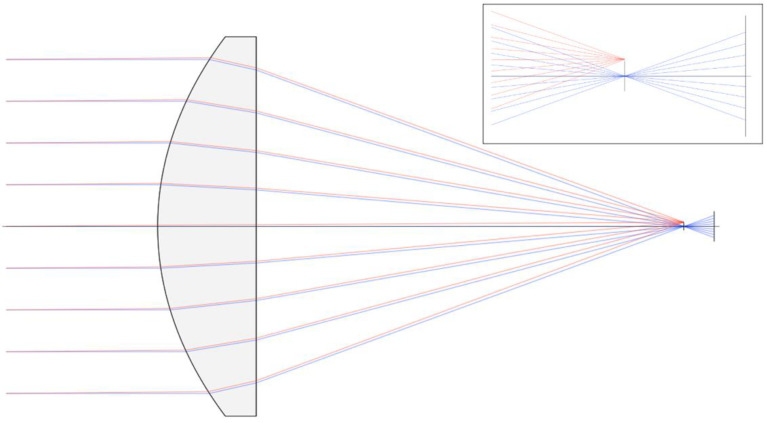
Ray-tracing model of the light path through the scanner telescopes (SWIR lens is shown). The vertical line at the lens focal plane represents either the MWIR sensor or the aperture in the SWIR channel. The far-right vertical line represents the SWIR sensor. Blue lines trace the path of incident light rays that are parallel to the lens axis. Red lines trace the path of light arriving from 0.5° off-axis. As shown, the 0.5° off-axis rays either miss the MWIR sensor or are blocked by the SWIR aperture (inset).

The optical configuration of both telescopes is shown in Figure 2. The incident light is focused to an aperture in the SWIR channel and directly onto the optical sensing element for the MWIR. The sizes of the SWIR aperture and MWIR optical sensing element are chosen such that only incident light within 0.5° of the lens axis will be detected, thus ensuring a 1° FOV.

The lenses, filters and sensors used in the two IR scanner channels are presented in Table 1. Both sensors have built-in thermoelectric coolers (TECs), and both are operated at a setpoint of −10 °C as a compromise between low noise, low power consumption and low heat dissipation. The narrowband filters are placed in front of the lens in both telescopes. A 13 mm entrance aperture is installed in the front of the SWIR telescope to reduce the incident light and prevent the SWIR sensor from saturating below 1500 °C. The entrance aperture of the MWIR telescope is 23 mm, essentially the size of the lens, for most effective light collection.

The 1.6 µm channel is sensitive to sunlight reflected by the ground surface. During daytime, variations in reflected sunlight due to changes in solar and observations angles, surface topography and albedo can cause significant shifts in background signal convolved with the fire signal. This shift cannot be quantified because the 1.6 µm albedo of the burning vegetation is unknown. Therefore, the 1.6 µm sensor is less accurate during daylight time for FRP measurement. The 4 µm channel is effectively solar blind, and therefore may be used for FRP measurement in both daytime and nighttime.

Both scanner channels have been calibrated over a temperature range of 200–1500 °C using a commercial blackbody source (Landcal Type R1500T, Land Instruments International). Based on these calculations, we report FRP expressed as blackbody equivalent fire temperature.

### 2.4. SWIR Camera

**Figure 3:**
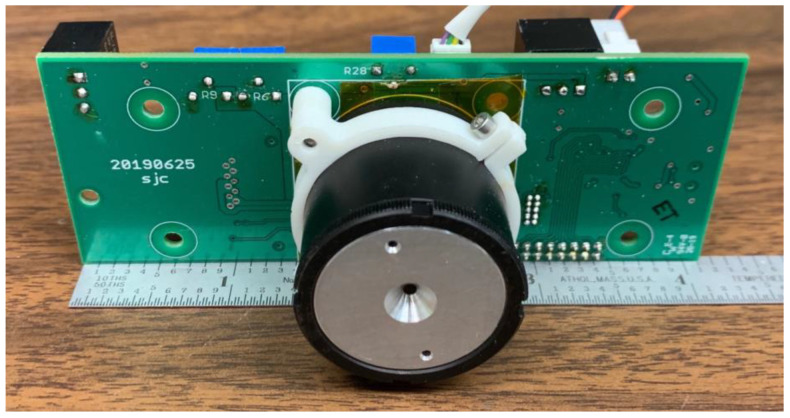
The AWSM custom-built SWIR camera.

**Figure 4:**
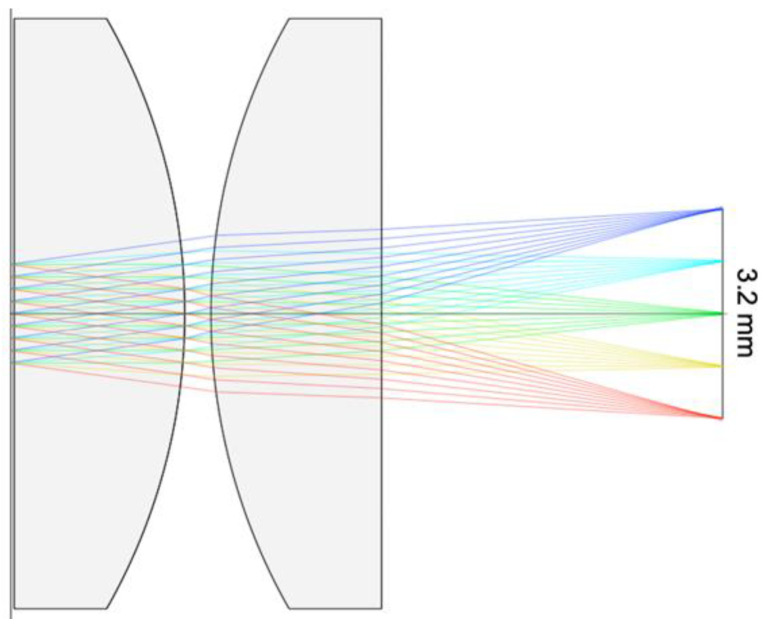
Ray-tracing model of the light path through the SWIR camera. The sensor array surface is represented by the thin vertical line on the right side. The red, yellow, green, teal and blue lines represent light incident on the lens surface at −13°, −6.5°, 0°, 6.5° and 13°, respectively. As shown, these rays are sharply focused on the sensor array surface except at the edges, with a linear image aberration less than 1.5%.

The custom SWIR camera (Figure 3) was designed for fire perimeter and extent measurements and to provide a consistency reference for fire FRP mapping using the scanning sensors. The camera uses a Hamamatsu G11097-0606S 64 × 64 element array image sensor with a 0.95–1.7 µm spectral response wavelength range. A pair of 9 mm lenses forms a simple light collector, which produces a 26° × 26° FOV with very little distortion (Figure 4). The angular resolution of the individual camera pixels is ~0.4° (26°/64). The camera is set to record images at a rate of 1 frame per second, which is sufficiently fast to provide overlapping images for full swath coverage during flight at design altitude and speed.

### 2.5. Visible/Thermal IR Dual Camera

The FLIR DUO/DUO-R visible/thermal dual camera (Teledyne FLIR, Wilsonville, OR) was selected for the AWSM instrument suite. This camera was chosen because of its compact size (41 × 59 × 30 mm), light weight (84 g) and reasonably low price (~USD 1000). The visible camera has 1920 × 1080 pixels with an 82° × 52° FOV. The thermal camera has 160 × 120 pixels, a sensing wavelength range of 7.5–13.5 µm, and a 57° × 44° FOV. The thermal camera saturates at approximately 380 °C. During AWSM operation, the dual camera records simultaneous visible and thermal images at 1 Hz, sufficient to produce overlapping images for full swath coverage.

### 2.6. Data System

Two Beagle Bone Black (BBB) industrial microcomputers (https://beagleboard.org/black, (accessed on 12 March 2023)) are employed for data acquisition and control of the AWSM instrument suite. One BBB is used to control the SWIR imager and the cross-track scanner and read their respective output data. The other BBB is used to read the data from the VectorNav VN-200. The time on the two BBBs is synchronized via an ethernet switch module (TE-202-003, SuperDroid Robots, Fuquay-Varina, NC, USA). As a result, all data read by both BBBs are synchronized. The FLIR Duo camera does not have a persistent internal clock or onboard storage and instead derives its file timestamp from the USB connection to the sensor BBB or another computer to which it is connected and where the files are saved. When a computer other than the sensor BBB is used, the fidelity of the synchronization with the BBBs is more difficult to maintain and additional effort in post-processing is required to align the images with the SWIR imager and scanner data.

## 3. Results

### 3.1. Small, Fixed-Wing UAS Deployment

**Figure 5:**
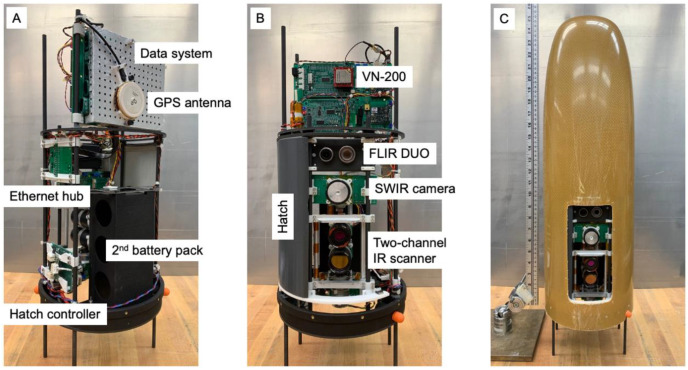
The AWSM sensors integrated into an S2 UAS payload frame. Top (**A**) and bottom (**B**) views of the AWSM system showing the arrangement of the individual sensors and components. Panel (**C**) shows the system inside the S2 nose cone with the hatch open. The hatch is closed before landing to protect the optics from debris.

The Black Swift Technologies (BST) S2 UAS (https://bst.aero/black-swift-s2-uas/, (accessed on 12 March 2023)) was selected as the operational platform for the NOAA NightFOX project. The S2 is a small, fixed-wing UAS that is versatile and has been used to deploy various types of scientific instrument payloads. The entire nose cone of the S2 is available payload space (20 cm ID × 50 cm L). A field-swappable payload system (Figure 5, S2 payload support structure with AWSM sensor suite and nose cone) allows easy integration of instruments and exchange of payloads between flights. Figure 5A,B show top and bottom views of the AWSM sensor suite, including all system components, configured as an S2 payload. The black enclosure visible in Figure 5A holds a second S2 battery pack (6S Li-ion, 22.8 V nominal, 14,000 mAh, 2.3 kg) that provides power both for the AWSM system and to extend the endurance of the S2. The entire payload as shown weighs just under 1500 g. The AWSM system power consumption during operation is approximately 15 W. In other applications, a 150 g Li-ion rechargeable battery pack (Tenergy 31012, 10.8 V, 2200 mAh) powers the instrument suite for over 2 h. Figure 5C shows the AWSM payload installed in an S2 nose cone. To simplify UAS operations in areas without smooth landing surfaces, and reduce flying weight, the S2 lands on its belly. Landing marks (scratches) on the nose cone are visible in Figure 5C. A hatch (dark grey piece visible in Figure 5B in the open position) seals the viewport opening prior to landing in order to protect the sensor suite.

At the AWSM design operational altitude of 1 km above ground level, the S2 would be safe from the heat of even very large wildfires.

**Figure 6:**
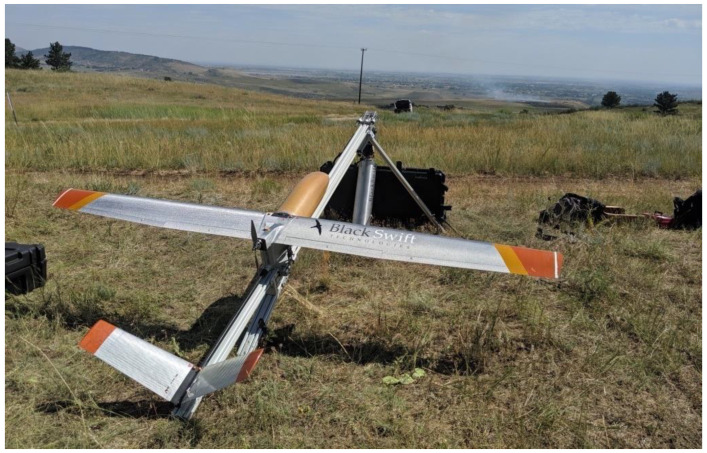
An S2 UAS carrying an AWSM payload ready for launch, Rabbit Mountain prescribed burn, Boulder County, Colorado, 31 July 2019.

The AWSM system configured for the S2 was tested during a flight opportunity over a small prescribed grassland burn in Boulder County, Colorado, on 31 July 2019. Figure 6 shows the AWSM-equipped S2 UAS mounted on a pneumatic launcher ready for deployment with smoke from the fire visible in the background.

**Figure 7:**
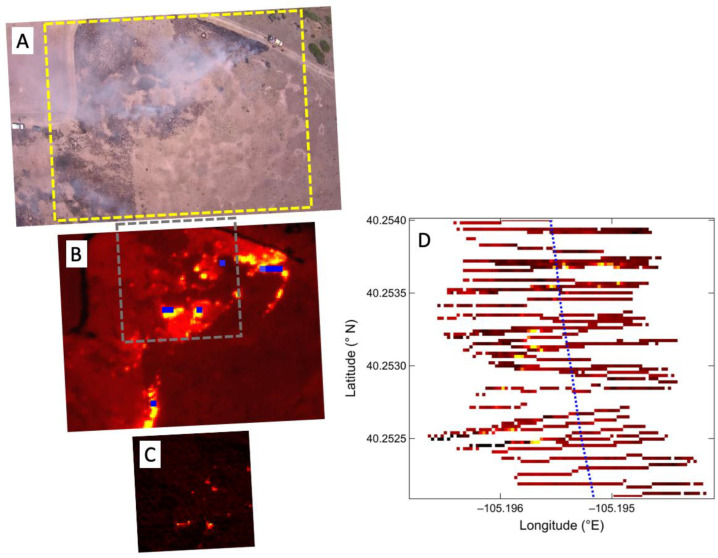
AWSM images and scanner data from an S2 UAS flight over a prescribed grassland burn during the active flaming period. The FLIR DUO visible wavelength camera (**A**) shows diffuse smoke across the area from multiple active fires. The yellow rectangle in (**A**) defines the area of the corresponding FLIR DUO thermal IR image shown in (**B**), where numerous fire spots are clearly visible across a burned area with elevated surface temperatures. The grey square in (**B**) defines the area of the approximately coincident SWIR camera image shown in (**C**). The blue dots in (**B**) are an overlay showing the location of fire pixels from the 4 um scanner channel. The 1.6 µm scanner channel measurement is shown in (**D**) and the sparse scanning resulting from the low altitude is apparent. The blue dotted line in (**D**) shows the flight track of the S2.

**Figure 8:**
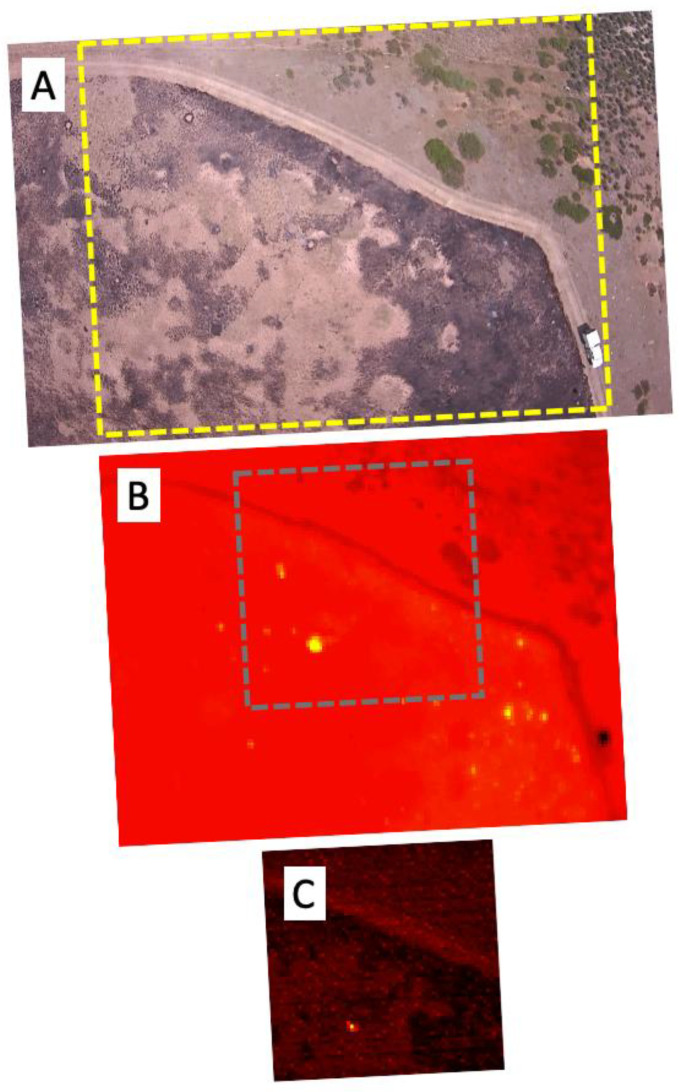
The same area of the prescribed grassland fire as shown in Figure 7, but in the post-active fire phase. No significant smoke is seen in the FLIR visible image (**A**), but the FLIR thermal camera (**B**) and custom SWIR camera (**C**) show distinct evidence of residual hot spots in the burned region. The yellow rectangle in (**A**) defines the area of the corresponding FLIR DUO thermal IR image (**B**), and the grey square in (**B**) defines the area of the approximately coincident SWIR camera image (**C**). The shift in the position of the SWIR camera image relative to the FLIR duo images from Figure 7 to Figure 8 is due to differences in the timing of image acquisition between the two cameras. The 1.6 µm scanner data are not shown because the lower temperatures were not distinguishable from the background created by scattered sunlight.

The S2 carrying the AWSM payload was flown during the prescribed burn with a 1.5-h endurance that allowed overflight during both the active flaming period as the fire line swept across the burn region and then again in the immediate post-flaming period. Examples of the imagery collected by the FLIR DUO visible and thermal IR cameras and the custom SWIR camera are shown in Figure 7 and Figure 8. Data from the 1.6 µm and 4 µm scanner channels are shown in Figure 7 when the active flaming yielded signals above detection limits. During these flights, the S2 was limited by US UAS operation regulations to a maximum altitude of 122 m above ground level, considerably lower than the 1000 m design altitude of the sensors. At the 18 m s^−1^ cruise speed of the S2, this resulted in incomplete spatial coverage, especially from the scanner, which at this altitude has a sample footprint of approximately two meters and the individual measurements yield a zigzag pattern of lines across the flight track (Figure 7D). The high degree of spatial correlation between the fires detected in the sparse scanner coverage and the fire spots in the thermal and SWIR images provides confidence in the geolocation of the scanner sample pixels.

The diffuse smoke present in the visible wavelength imagery during the active burning phase (Figure 7A) is absent from the visible imagery in the post-fire phase (Figure 8A). The thermal IR and SWIR imagery reveal the presence of a number of residual hot spots, demonstrating the sensitivity of these channels to lower temperatures.

### 3.2. NOAA Twin Otter Deployment

**Figure 9:**
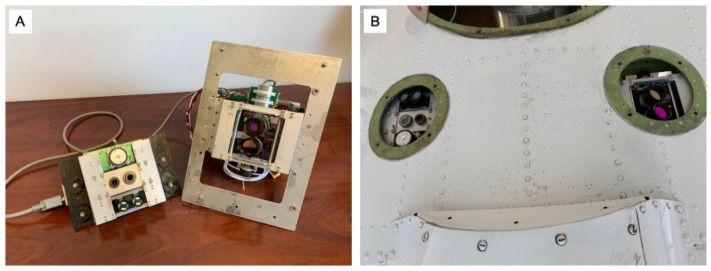
For integration on an NOAA Twin Otter aircraft, the AWSM instrument suite was split into two parts (**A**) and installed to use two 11 cm diameter ports (**B**) in the aft payload bay.

The AWSM sensor suite configuration is highly flexible, allowing the instrument layout to be rearranged for integration onto different platforms. The AWSM system was flown onboard the NOAA-Met Twin Otter aircraft as part of the NOAA/NASA FIREX-AQ mission (https://csl.noaa.gov/projects/firex-aq/, (accessed on 12 March 2023)) in July and August 2019. To make use of two small (11 cm diameter) open viewports in the aft payload bay, the ASWM sensor suite was split into two separate components (Figure 9A), with the cross-track scanner occupying one viewport and the SWIR and FLIR cameras the other (Figure 9B). Furthermore, onboard the aircraft was a microJoule class scanning Doppler lidar [20]. The lidar was used to measure horizontal wind fields and fire plume dynamics and provided a continuous measurement of aircraft height AGL.

During the FIREX-AQ mission, the Twin Otter conducted flights over a number of small fires in Idaho and Oregon. The aircraft typically flew at altitudes between 1.2 and 3.6 km AGL. The resulting measurement footprints of the scanning telescopes on ground were between 21 and 80 m in diameter, depending on the viewing angle. The AWSM payload completed 6 successful flights during the mission [21].

**Figure 10:**
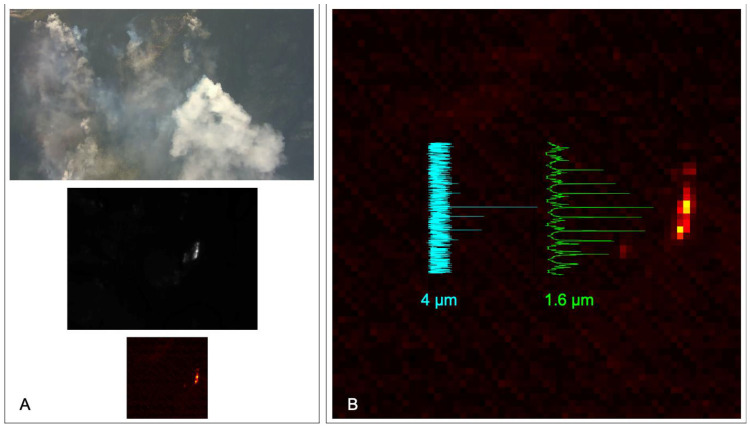
Images and scanner data of the Barren Hill wildfire in Idaho on 29 July 2019 observed from the NOAA-Met Twin Otter. (**A**) Images from the FLIR visible (**top**) and thermal (**middle**) cameras and SWIR camera (**bottom**). (**B**) The SWIR camera image is the same as shown in Panel A. Corresponding time series of the telescope scanner data (green = 1.6 µm, cyan = 4 µm) are also shown. The scanner time (vertical) axis is scaled to match the camera image.

One of the wildfires overflown by the NOAA-Met Twin Otter during FIREX-AQ is shown in Figure 10. FLIR visible and thermal images and the SWIR image are shown in Figure 10A. The same SWIR image with overlaid raw SWIR and MWIR telescope scanner data is shown in Figure 10B. As mentioned previously, the MWIR sensor is only sensitive to average FOV temperatures above 320 °C. The higher absolute sensitivity of the SWIR sensors is apparent in the greater number of fire pixels detected.

**Figure 11:**
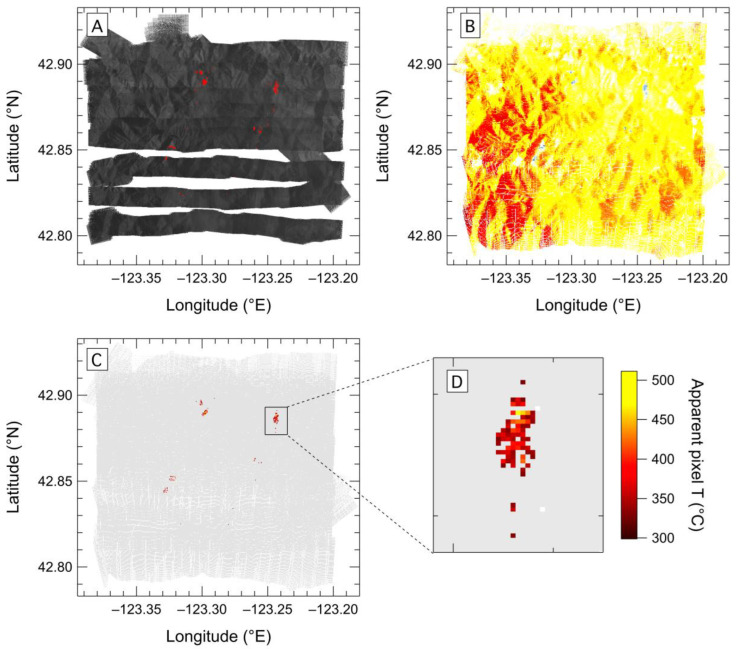
Maps of the Milepost 97 fire in Oregon on 27 July 2019 produced using the (**A**) SWIR camera, (**B**) 1.6 µm scanner channel and (**C**,**D**) 4 µm scanner channel. The flight took place during daytime, so terrain is clearly visible in the both of the SWIR sensor (**A**,**B**) maps. In panel (**A**), fire containing pixels are shown in red superimposed on a greyscale map. (**A**) color scale is used in (**B**) to highlight the contrast in the terrain-reflected sunlight. Detected fire locations appear as blue pixels. The 4 µm sensor (**C**,**D**) is blind to reflected sunlight, so non-fire pixels have a uniform background signal intensity (light grey), while fire pixels are clearly visible and the apparent temperature of the fire pixels is represented by the color scale.

Figure 11 presents an example of the mapping capability of the AWSM package from the Twin Otter sortie over the Milepost 97 fire in Oregon on 27 July 2019. Maps have been constructed using data from the SWIR camera and the two IR scanner channels. Because the data were acquired during daytime, terrain features are clearly visible in the SWIR camera and scanner maps. The MWIR scanner is solar blind, so terrain is not visible and the fire pixels are clearly discernable from the uniform background. In a magnified view of one of the active fire regions, the average temperature of individual fire-containing pixels ranged from the 320 °C detection limit up to 500 °C.

Images from the SWIR camera and the scanning sensor data were synchronized with the VectorNav VN-200 aircraft position and attitude data, allowing exact ground location to be determined, and could therefore be used to produce fire or fire radiative power maps. The FLIR Duo camera in the FIREX-AQ configuration was connected to a separate computer for real-time display. As a result, the FLIR images were not synchronized with the VN-200 data and could not be used to make corresponding accurate visible and thermal maps.

### 3.3. Uncertainties in Fire Temperature Measurements

The uncertainty in the determination of fire temperature from the 4 µm sensor arises primarily from the noise in the sensor circuit. As shown in Figure 10B, the background (fire-free) noise band is about 0.01 V peak-to-peak and the corresponding standard deviation is about 0.0034 V. This precision translates to an uncertainty of ±10 °C at the minimum detectable temperature of 320 °C, at which the sensor signal is about 0.011 V (3σ) above the mean background. This relative uncertainty reduces quickly with increase in pixel temperature, as the signal grows exponentially with fire temperature.

There are a few possible approaches to reducing the uncertainty of the 4 µm sensor. The design of the sensor circuit might be improved and better shielded to attempt to reduce electronic noise. The inherent performance of the sensing element (Table 1) depends on its operation temperature. Here the sensor temperature was set at −10 °C, which can be reduced to about −20 °C for higher sensitivity and lower noise, but at the cost of requiring additional power and heat dissipation. Optically, the telescope diameter could be expanded to increase the absolute signal level.

The signal-to-noise ratio of the 1.6 µm sensor is high enough that the intrinsic background noise does not cause a significant uncertainty in fire temperature measurement. However, as noted above, this telescope is sensitive to solar light reflected by the terrain (Figure 9B), which produces significant variations in the background that contribute to uncertainty in the measurement during daytime.

As noted above, the reported fire temperature is a measurement of fire radiative power expressed as the temperature of a blackbody that emits the same amount of energy in the sensor’s wavelength band.

### 3.4. Uncertainties in Fire Mapping

Two major sources of error are involved in translating the scanning sensor and camera data into maps. The first uncertainty is caused by pointing errors. The optical arrangements shown in Figure 2 and Figure 4 were idealized, with the lens(es) and detector aligned perfectly. In reality, small offsets in component alignment and lens tilting are inevitable, which result in a pointing error. This error is mechanically fixed and may be removed via careful calibration. We have yet to perform this calibration. The error in telescope pointing is estimated to be less than 3° based on repeated observations of a fire from different aircraft orientations (e.g., Figure 11D showing overlapping points from passes in opposite directions). A calibration fixture using a fixed IR point source could be used to better assess the pointing error for data correction in post-processing.

The second source of error is caused by the relative position of the aircraft to the ground, which is represented by AGL. The micropulsed Doppler lidar [18] onboard the Twin Otter provided accurate AGL values. The S2 UAS payload does not include a lidar, so the accuracy of AGL value at a given point is dependent on the accuracy of the digital elevation. Furthermore, the mapping software currently assumes a flat surface. This assumption is certainly not good in mountainous regions (Figure 11). In the future, topographic maps may be incorporated into the mapping software to reduce the resulting error.

## 4. Conclusions

A miniature remote sensing instrument suite, AWSM, with three optical instruments covering four wavelength bands for wildfire FRP measurement and fire extent mapping has been designed, constructed and flown onboard a small UAS and a crewed aircraft. The goal of the project was to develop an instrument package that is compact, lightweight and relatively inexpensive, with low power consumption, suitable for small UAS deployment, yet is sufficiently sensitive to provide a research quality complement to satellite and large research-grade airborne instruments. The AWSM payload system is highly configurable and could be easily adapted for use on a number of different aerial platforms such as multirotor drones without significant modification. The ultimate goal is to develop a sensor suite that could be widely adopted for wildfire incident command situational awareness and fire weather model input data collection with small UASs and light crewed aircraft.

The completed AWSM package meets the design criteria of being light weight (1500 g), sensitive (minimum pixel average FRP detection temperature of 320 °C), low power (15 W) and relatively low cost (~USD 11 k excluding labor). The most important applications of the package are fire area mapping and fire radiative power measurement. At the design operational altitude of 1 km AGL, the spatial resolution for these two functions are approximately 7 m and 18 m, respectively. These values are comparable to those from much more sophisticated, large scientific instruments such as MASTER flown on the NASA DC-8 aircraft at the typical cruise altitude of ~12 km.

The AWSM package is very robust despite its appearance. Onboard the S2 UAS, the package endured multiple 10 g launches and belly-skid landings hard enough to crack a carbon fiber nose cone without experiencing any performance degradation. Being lightweight, robust and relatively inexpensive, AWSM is ideal for UAS platforms and light manned aircraft for routine operations.

## Figures and Tables

**Table 1:** Components of the scanning telescope and SWIR camera.

	Sensor	Lens	Filter
Scanning telescope (4 µm)	Teledyne Judson Technologies J23TE2-66C-R500U-1.9	Edmund Optics #68-247	Spectrogon NB-3960-070
Scanning telescope (1.6 µm)	Hamamatsu P13243-122MS	Edmund Optics #47-731	Spectrogon BP-1610-060
SWIR camera	Hamamatsu G11097-0606S	Edmund Optics #32-009	No filter

## Data Availability

AWSM data from the from the Rabbit Mountain prescribed burn S2 UAS flight (Section 3.1) is available online at http://csl.noaa.gov/projects/nightfox/data/ (accessed on 12 March 2023) and data from the Twin Otter deployment during the FIREX-AQ mission (Section 3.2) is available online at https://csl.noaa.gov/groups/csl7/measurements/2019firex-aq/TwinOtterMET/ (accessed on 12 March 2023).
